# Modified Early Warning Score as a predictor of intensive care unit readmission within 48 hours: a retrospective observational study

**DOI:** 10.5935/0103-507X.20200047

**Published:** 2020

**Authors:** Ahmed Naji Balshi, Basim Mohammed Huwait, Alfateh Sayed Nasr Noor, Abdulrahman Mishaal Alharthy, Ahmed Fouad Madi, Omar Elsayed Ramadan, Abdullah balahmar, Huda A. Mhawish, Bobby Rose Marasigan, Alva Minette Alcazar, Muhammad Asim Rana, Waleed Tharwat Aletreby

**Affiliations:** 1 Critical Care Department, King Saud Medical City - Riyadh, Saudi Arabia.; 2 Anesthesia Department, Faculty of Medicine, Tanta University - Tanta, Egypt.; 3 Anesthesia Department, Faculty of Medicine, Ain Shams University - Cairo, Egypt.; 4 Internal Medicine and Critical Care Department, Bahria Town International Hospital -Lahore, Pakistan.

**Keywords:** MEWS, Patient readmission, Intensive care units, Critical care, Length of stay, MEWS, Readmissão do paciente, Unidades de terapia intensiva, Cuidados críticos, Tempo de permanência

## Abstract

**Objective:**

To evaluate the hypothesis that the Modified Early Warning Score (MEWS) at the time of intensive care unit discharge is associated with readmission and to identify the MEWS that most reliably predicts intensive care unit readmission within 48 hours of discharge.

**Methods:**

This was a retrospective observational study of the MEWSs of discharged patients from the intensive care unit. We compared the demographics, severity scores, critical illness characteristics, and MEWSs of readmitted and non-readmitted patients, identified factors associated with readmission in a logistic regression model, constructed a Receiver Operating Characteristic (ROC) curve of the MEWS in predicting the probability of readmission, and presented the optimum criterion with the highest sensitivity and specificity.

**Results:**

The readmission rate was 2.6%, and the MEWS was a significant predictor of readmission, along with intensive care unit length of stay > 10 days and tracheostomy. The ROC curve of the MEWS in predicting the readmission probability had an AUC of 0.82, and a MEWS > 6 carried a sensitivity of 0.78 (95%CI 0.66 - 0.9) and specificity of 0.9 (95%CI 0.87 - 0.93).

**Conclusion:**

The MEWS is associated with intensive care unit readmission, and a score > 6 has excellent accuracy as a prognostic predictor.

## Abstract (pt)

**Objetivo:**

Avaliar a hipótese de que o *Modified Early Warning Score* (MEWS) por ocasião da alta da unidade de terapia intensiva associa-se com readmissão, e identificar o nível desse escore que prediz com maior confiabilidade a readmissão à unidade de terapia intensiva dentro de 48 horas após a alta.

**Métodos:**

Este foi um estudo observacional retrospectivo a respeito do MEWS de pacientes que receberam alta da unidade de terapia intensiva. Comparamos dados demográficos, escores de severidade, características da doença crítica e MEWS de pacientes readmitidos e não readmitidos. Identificamos os fatores associados com a readmissão em um modelo de regressão logística. Construímos uma curva Característica de Operação do Receptor para o MEWS na predição da probabilidade de readmissão. Por fim, apresentamos o critério ideal com maior sensibilidade e especificidade.

**Resultados:**

A taxa de readmissões foi de 2,6%, e o MEWS foi preditor significante de readmissão, juntamente do tempo de permanência na unidade de terapia intensiva acima de 10 dias e traqueostomia. A curva Característica de Operação do Receptor relativa ao MEWS para predizer a probabilidade de readmissão teve área sob a curva de 0,82, e MEWS acima de 6 teve sensibilidade de 0,78 (IC95% 0,66 - 0,9) e especificidade de 0,9 (IC95% 0,87 - 0,93).

**Conclusão:**

O MEWS associa-se com readmissão à unidade de terapia intensiva, e o escore acima de 6 teve excelente precisão como preditor prognóstico.

## INTRODUCTION

Readmission to the intensive care unit (ICU) has been linked in the literature to poor patient outcomes, including higher mortality and longer length of stay.^(1)^-^(3)^ Additionally, readmission to the ICU imposes financial burdens and causes patient flow inefficiencies on healthcare systems.^(1,4)^ There is general agreement that the decision to discharge patients from the ICU is a subjective judgment of the attending intensivist based entirely on clinical assessments.^(3,5)^ However, several other nonclinical factors come into play in making such a decision, such as high demand and need for ICU beds by emergency departments and operative theaters,^(6,7)^ thus rendering the discharge decision a complex, challenging, and high-risk transition of care process.^(5)^ These factors may result in the premature and suboptimal discharge of patients,^(8)^ which escalates the risk of readmission, since up to 42% of prematurely discharged patients eventually end up readmitted to the ICU.^(2)^

Several efforts have been put forward to optimize and prioritize ICU discharges, either by identifying risk factors associated with ICU readmission^(9,10)^ or developing readmission prediction models.^(11,12)^ Unfortunately, very few risk stratification models have ever been validated, and their reliability is questionable; furthermore, it is not clear how these models compare to other methods or if they provide additional value over the clinical judgment of physicians in identifying readiness to be discharged from the ICU.^(5,13,14)^

Early this century, the Modified Early Warning Score (MEWS) was developed in the United Kingdom in response to audit commission recommendations to aid ward staff in detecting deteriorating patients who may need ICU or high-dependency unit admission. Different models of the MEWS with minor differences are currently being used in many countries, including the United States, Australia, and the Netherlands, while in the United Kingdom, the MEWS is a mandatory standard of care, possibly due to its simple, easy, and rapid nature.^(15)^ Very closely related models exist with other names, such as “Code Yellow” in Brazil.^(16)^

The scoring system adopted by our institute (King Saud Medical City) consists of 7 physiological parameters (systolic blood pressure, respiratory rate, heart rate, temperature, oxygen saturation, oxygen supplementation, and level of consciousness). A score is given to different ranges of each variable, and the sum of these scores constitutes the final score (Table 1).

**Table 1:** Modified Early Warning Score

Physiological parameter	Score						
	3	2	1	0	1	2	3
Respiratory rate (bpm)	< 8		9 - 11	12 - 20		21 - 29	> 30
SpO_2_ (%)	< 91	92 - 93	94 - 95	> 96			
Oxygen supplement		Yes		No			
Systolic blood pressure (mmHg)	< 90	91 - 100	101 - 110	111 - 200		200 - 219	> 220
Heart rate (bpm)	< 40		41 - 50	51 - 100	101 - 110	111 - 130	> 131
Temperature (ºC)		< 35	35.1 - 36	36.1 - 38	38.1 - 39	> 39.1	
Consciousness				A	V	P	U

SpO_2_ - oxygen saturation; A - alert; V - responds to verbal stimuli; P - responds to painful stimuli; U - unresponsive.

This study was conducted to evaluate the hypothesis that the MEWS at the time of ICU discharge is associated with readmission and to identify the MEWS that most reliably predicts ICU readmission within 48 hours of discharge.

## METHODS

This was a retrospective chart review cohort study carried out in the mixed adult ICU at King Saud Medical City (KSMC), Riyadh, Saudi Arabia. King Saud Medical City is a tertiary referral hospital and the largest government hospital in Saudi Arabia (1,200 beds). The ICU has a total of 127 beds and an average bed occupancy rate of 95%. This is a closed ICU operated 24/7 by in-house intensivists. Patient management generally follows international guidelines and protocols for standardized management. The ICU is divided into several units, including medical, surgical, neurocritical, trauma, burn and maternity units (latter two were not included in the study). The physician-to-patient ratio is 8, while the nurse-to-patient ratio is 1. As a tertiary referral center, transfers to other healthcare facilities are rare (approximately 0.5% of all discharges) and are usually to rehabilitation centers for chronic patients with prolonged stays who are unfit for discharge to the general ward. Discharged patients from the ICU are transferred to their respective general wards, as our institute lacks a step-down unit. Hence, it is the policy of the Rapid Response Team (RRT) to follow all patients discharged from the ICU for 48 hours to identify deteriorating patients who require ICU readmission in a timely fashion.

The MEWS was calculated for all patients who were alive at discharge from the ICU between June 1^st^, 2018 and May 31^st^, 2019. Subsequently, all patients were followed for 48 hours to identify the readmitted patients. All discharges were planned by the attending intensivist during the morning or night round.

The following patients were excluded from our study: patients aged younger than 18 years, maternity and burn ICU patients, patients who stayed in the ICU for less than 48 hours (such as postoperative patients), patients discharged home or to another healthcare facility directly from the ICU or within 48 hours from ICU discharge, patients who died in the ICU, and patients discharged with Do Not Resuscitate orders. If a patient was admitted to the ICU more than once during the study period, only the key admission was considered.

The study was approved by our institutional review board with waiver of consent *(*Ref. number H1RI-11-Sep19-01*)* and observes the ethical principles outlined by the Helsinki declaration.

We collected all pertinent demographic data (age, sex, diagnosis, Acute Physiology and Chronic Health Evaluation (APACHE) 4 score upon ICU admission, mechanical ventilation status, and requirement for vasopressor agents upon ICU admission) on a predesigned spreadsheet. A trained nurse calculated and recorded the MEWS, in addition to Sequential Organ Failure Assessment (SOFA) score, tracheostomy status, continuous renal replacement therapy (CRRT) status, ICU length of stay, and Glasgow Coma Scale, just as the patient was being discharged out of the ICU. Patients who were readmitted to the ICU within 48 hours and the causes of readmission were noted.

### Statistical method

Continuous data are presented as the mean ± standard deviation (SD) and were compared by Student’s t-test or the Mann-Whitney U test as appropriate according to the Shapiro-Wilk test of normality. Categorical data are presented as numbers (%) and were compared by the chi-square test or Fisher’s exact test as appropriate. When a t-test was used, we reported the p value of the Wald test to account for unequal variance.

Univariable logistic regression was used to identify variables associated with readmission; consequently, a multivariable logistic regression model including all variables with a p-value < 0.1 in the univariable analysis was fitted using the backward elimination stepwise method, and the result was presented as an odds ratio (OR) with a corresponding 95% confidence interval (95%CI). The goodness of fit for the model was evaluated by the Hosmer-Lemeshow test (considered well fitted with a p-value > 0.05). The collinearity of continuous predictors was evaluated using the variance inflation factor (VIF), and any variable in the model with an VIF > 5 was removed.

Thereafter, we constructed a receiver operator characteristics (ROC) curve and presented the area under the curve (AUC). The sensitivity, specificity, positive predictive value, and negative predictive value of the optimum criterion were similarly presented with 95%CI.

All statistical tests were two tailed, and a p-value < 0.05 was considered significant. Statistical tests were conducted using commercially available software (StataCorp. 2017. *Stata Statistical Software: Release 15*. College Station, TX: StataCorp LLC).

## RESULTS

**Figure 1:**
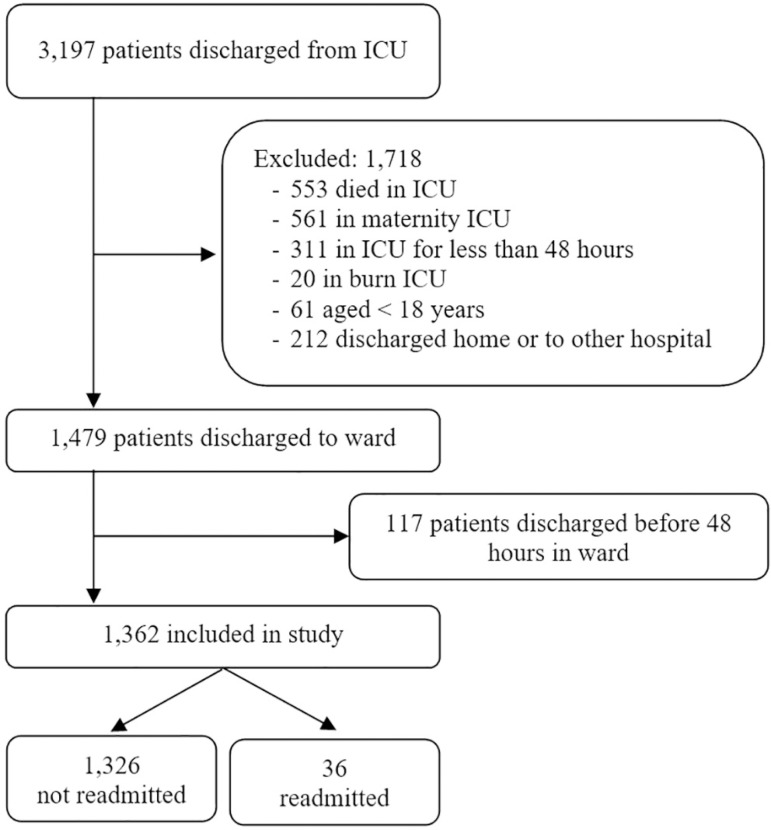
Flowchart of the study for patient inclusion.

We discharged 3,197 patients from the ICU between June 1^st^, 2018 and May 31^st^, 2019. A total of 1,718 patients were not eligible for the study, while the MEWS was calculated upon discharge to the ward for 1,479 patients. Another 117 patients were excluded because they were discharged from the hospital within 48 hours of ICU discharge, leaving a total of 1,362 patients who were included in the study (Figure 1). All eligible patients were included in the analysis without any loss of follow-up.

ICU - intensive care unit.

**Figure 2:**
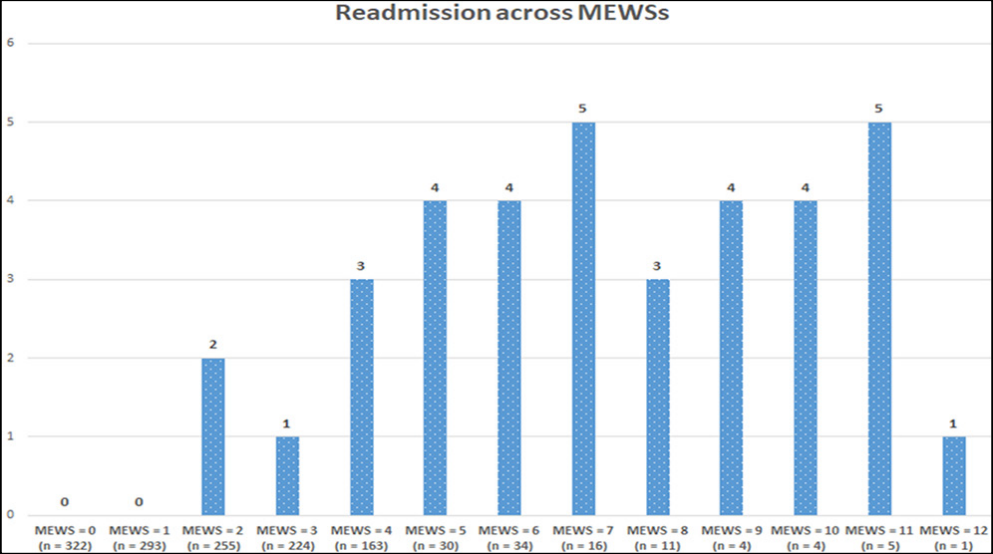
Number of readmitted patients with each Modified Early Warning Score.

There were 36 cases of readmission to the ICU within 48 hours of discharge (2.6%), and the patients who were readmitted were comparable to those who were not in regards to age, sex, APACHE 4 admission score, mechanical ventilation upon admission, need for CRRT, diagnostic category, and hospital mortality. However, there was a statistically significant difference between the two groups concerning the MEWS, ICU length of stay, Glasgow Coma Scale and SOFA scores at discharge, and frequency of tracheostomy (Table 2). Readmitted patients had a mean MEWS of 6.8 ± 2.8, whereas those who were not readmitted had a mean score of 2.5 ± 2.1 (p < 0.001). Figure 2 depicts the number of readmitted patients across different MEWSs. The most commonly identified cause of readmission was respiratory failure (56%, 20 patients); within this category, patients were readmitted due to tachypnea, desaturation, high oxygen requirement, and an immediate or anticipated need for endotracheal intubation and mechanical ventilation. The next most common category was hemodynamic instability (31%, 11 patients), in which patients became hypotensive, tachycardic, and required vasopressor support despite adequate fluid resuscitation. Finally, 12% (5 patients) were readmitted due to deterioration of consciousness, requiring airway protection. Of the 36 readmitted patients, 15 were later identified in the ICU to be septic based on positive cultures.

**Table 2:** Demographic and clinical characteristics

	Not readmitted within 48 hours	Readmitted within 48 hours	p value
	(n = 1326)	(n = 36)	
Age	46 ± 18.5	45.3 ± 14.5	0.9*
Males	948 (71)	26 (72)	0.8
APACHE 4	69.4 ± 12.3	72.1 ± 11.7	0.4*
MV upon admission	657 (49.5)	20 (56)	0.5
Required vasopressors	582 (44)	17 (47)	0.9
Diagnosis			
Medical	610 (46)	18 (50)	0.8
Surgical	557 (42)	14 (39)	0.9
Trauma	159 (12)	4 (11)	0.9
SOFA score at discharge	5.9 ± 1.5	6.8 ± 1.4	0.01^†^
ICU length of stay	13.3 ± 15.2	22.5 ± 44.4	0.02^†^
Tracheostomy	66 (5)	11 (31)	< 0.001
CRRT	289 (22)	10 (28)	0.5
GCS at discharge	12.9 ± 1.8	9.9 ± 1.7	<0.001^†^
MEWS	2.5 ± 2.1	6.8 ± 2.8	< 0.001^†^
Hospital mortality	212 (16)	10 (27.8)	0.09

APACHE - Acute Physiology and Chronic Health Evaluation; MV - mechanical ventilation; SOFA - Sequential Organ Failure Assessment; ICU - intensive care unit; CRRT - continuous renal replacement therapy; GCS - Glasgow Coma Scale; MEWS - Modified Early Warning Score.

* Student's t-test;

† Mann-Whitney U test. Results expressed as mean ± standard deviation or n (%).

MEWS - Modified Early Warning Score.

Five variables had p values < 0.1 in univariable logistic regression. However, with only 36 events, we could enter 3 - 4 variables at most into the multivariable logistic regression to avoid overfitting, and since Glasgow Coma Scale is actually one of the variables used to calculate MEWS, it was excluded. Furthermore, ICU length of stay was entered in the model as a dichotomized variable of ≤ 10 days and > 10 days since the data were highly skewed.^(17)^ Accordingly, the variables in the model were the SOFA score at discharge, dichotomized ICU length of stay, tracheostomy, and MEWS. The multivariable logistic regression model identified three variables as significant for ICU readmission: higher MEWS upon discharge (for each 1-point increase, OR = 1.5; 95%CI 1.2 - 1.8; p < 0.001), presence of tracheostomy (OR = 13.4; 95%CI 4.4 - 40.1; p < 0.001), and ICU length of stay > 10 days (OR = 5.7; 95%CI 1.7 - 18.5; p = 0.004). The SOFA score at discharge was not statistically significant (OR = 1.3; 95%CI 0.94 - 1.7; p = 0.13) (Table 3).

**Table 3:** Risk factors for intensive care unit readmission within 48 hours of intensive care unit discharge

	Uni-variable			Multivariable		
	OR	95%CI	p value	OR	95%CI	p value
MEWS	1.6	1.3 - 1.9	< 0.001	1.5	1.2 - 1.8	< 0.001
SOFA score	1.5	1.01 - 2	0.01	1.3	0.94 - 1.7	0.1
Length of stay > 10 days	3.9	1.4 - 10.7	0.03	5.7	1.7 - 18.5	0.004
Tracheostomy	14.3	5.6 - 36.2	< 0.001	13.4	4.4 - 40.1	< 0.001

OR - odds ratio; 95%CI - 95% confidence interval; MEWS - Modified Early Warning Score; SOFA - Sequential Organ Failure Assessment. Multivariable model calibration: Hosmer-Lemeshow; p = 0.95; area under the curve = 0.82. Variable inflation factor: MEWS = 1.04.

The ROC curve of the MEWS for predicting the risk of readmission was drawn and had an AUC of 0.82 (95%CI 0.78 - 0.84; p value of Z statistic < 0.001). The analysis of the different cut off MEWSs identified a score of more than 6 as the optimum criterion that yields the best combination of sensitivity and specificity. The identified optimum criterion (more than 6) yielded a sensitivity of 0.78 (95%CI 0.66 - 0.9), specificity of 0.9 (95%CI 0.87 - 0.93), positive predictive value of 0.19 (95%CI 0.11 - 0.29), and a negative predictive value of 0.99 (95%CI 0.981 - 0.997).

## DISCUSSION

In this study, 1,362 discharged patients were included in the analysis for the period between June 1^st^, 2018, and May 31^st^, 2019, out of a total 3,197 patients discharged from the ICU. Out of the included population, 36 patients were readmitted to the ICU within 48 hours, with a readmission rate of 2.6% for at-risk patients. Even though actual readmission rate calculations account for all ICU discharges within a period of time,^(1)^ this rate remains comparable to the readmission rates reported in many studies.^(4,14,18)^ Group comparisons revealed that both groups were similar in terms of age, sex, APACHE 4 score, general diagnostic category (medical/surgical/trauma), ventilation status, vasopressor requirement upon admission, and the need for CRRT upon ICU discharge. Similar findings have also been reported in other studies with regard to sex distribution,^(19,20)^ APACHE 2 score upon admission,^(18,21)^ and all three variables (age, sex and APACHE socore).^(22)^ Although our research group has previously^(3)^ identified the APACHE 4 score as a risk factor for ICU readmission and found that it was significantly different between readmitted and not readmitted patients, the definition of ICU readmission in that study was at any time during the key hospitalization. The variables that were different between groups in this study were the MEWS upon ICU discharge, ICU length of stay, tracheostomy, SOFA score and Glasgow Coma Scale at discharge. When entered into a well-fitted logistic regression model, the MEWS, dichotomized ICU length of stay, and tracheostomy status retained their significance. This finding coincides with that of many similar studies. Klepstad et al.^(19)^ reported that a very similar score (National Early Warning Score - NEWS) was the only predictor of ICU readmission. The NEWS was also an independent predictor of ICU readmission - in addition to acute renal failure - in the work by Uppanisakorn et al.^(22)^ The work by Reini et al.^(23)^ is in contrast to these findings. In their study, the MEWS at ICU discharge was not a predictor of ICU readmission (OR = 0.98; 95%CI 0.69 - 1.37); however, this result might be influenced - as acknowledged by the authors - by the decision to withhold ICU readmission for 10 out of 15 patients discharged with a MEWS of 5 or more.

Despite being originally developed to assist the staff of medical or surgical wards in identifying clinically deteriorating patients^(15)^ and being validated for that role,^(24)^ the MEWS has been studied extensively as a predictor of ICU readmission with variable results. However, studies providing details of the sensitivity and specificity of a specific cutoff MEWS as a prognostic predictor of readmission are scarce. In our study, the ROC curve of the risk of readmission had an area under the curve of 0.82 (95%CI 0.78 - 0.84; p-value of Z statistic < 0.001), and the optimum criterion as a readmission prognostic predictor was a MEWS of more than 6. A MEWS > 6 was highly specific (0.9; 95%CI 0.87 - 0.93), with a lower sensitivity (0.78; 95%CI 0.66 - 0.9), a positive predictive value of 0.19 (95%CI 0.11 - 0.29), and a negative predictive value of 0.99 (95%CI 0.981 - 0.997). These results indicate that when the MEWS is higher than 6, the likelihood of readmission is very high (90%), and when the MEWS is 6 or less, the patient will not be readmitted in 78% of the cases. Similar reported results^(21)^ show an area under the curve of 0.93, sensitivity of 92%, and a specificity of 85% for a cutoff MEWS > 7, and although this study shows the excellent prognostic capabilities of the MEWS, the differences with our results may have been due to the consideration of readmission within only 24 hours of ICU discharge; considering readmission within 48 hours gives an extra 24 hours for patients with a slightly lower MEWS to deteriorate and be readmitted to the ICU, which was accounted for in our study. In contrast to these results is the lower area under the curve of only 0.6 (95%CI 0.58 - 0.62) for the MEWS in the work by Rojas et al.^(25)^ It must be noted, however, that they considered a longer period of readmission of up to 7 days.

It is imperative as we present the results of our study to acknowledge that the Society of Critical Care Medicine (SCCM) recommends against discharging patients from the ICU based on scores of illness severity,^(26)^ and as we endorse this recommendation, we must stress upon the fact that ICU discharge may be hastened by the need for ICU beds; hence, we advocate for determining the MEWS at ICU discharge merely as an assistive tool to make a better-informed decision. While observing the recent emerging evidence that ICU readmission itself may not be a risk factor for mortality thereafter, which precludes the rationale of using readmission rates as quality indicators,^(27)^ this can be observed in the comparable hospital mortality rates in our study.

Our study suffers from the inherent limitations of its retrospective observational design, the most obvious of which is the imbalance between groups. Furthermore, we failed to evaluate several factors that have been linked in the literature to ICU readmission, such as the source of ICU admission, postoperative status, time and day of ICU discharge, and comorbidities.^(9,11,12)^ We did not evaluate the different components of the MEWS; should we have performed such an analysis, we might have identified the components most associated with readmission. We also did not evaluate readmission rates across different MEWSs. Furthermore, we only evaluated readmission within 48 hours, not accounting for patients readmitted after that period. Moreover, a survival analysis based on the duration until readmission may have yielded more informative results. Finally, in the logistic regression model, the linearity of independent variables and log odds was assumed, which may not hold true.

## CONCLUSION

The Modified Early Warning Score at intensive care unit discharge is an independent predictor of intensive care unit readmission within 48 hours. A score greater than 6 has an excellent accuracy in predicting intensive care unit readmission with an area under the curve of 82% and may be useful in identifying patients at a greater risk of intensive care unit readmission.
